# Dose-dense sequential adjuvant chemotherapy followed, as indicated, by trastuzumab for one year in patients with early breast cancer: first report at 5-year median follow-up of a Hellenic Cooperative Oncology Group randomized phase III trial

**DOI:** 10.1186/1471-2407-14-515

**Published:** 2014-07-15

**Authors:** George Fountzilas, Urania Dafni, Christos Papadimitriou, Eleni Timotheadou, Helen Gogas, Anastasia G Eleftheraki, Ioannis Xanthakis, Christos Christodoulou, Angelos Koutras, Christos N Papandreou, Pavlos Papakostas, Spyros Miliaras, Christos Markopoulos, Constantine Dimitrakakis, Panagiotis Korantzopoulos, Charisios Karanikiotis, Dimitrios Bafaloukos, Paris Kosmidis, Epaminontas Samantas, Ioannis Varthalitis, Nicholas Pavlidis, Dimitrios Pectasides, Meletios-Athanassios Dimopoulos

**Affiliations:** 1 Department of Medical Oncology, “Papageorgiou” Hospital, Aristotle University of Thessaloniki School of Medicine, Thessaloniki Ring Road, 564 03 Thessaloniki, Macedonia, Greece; 2 Laboratory of Biostatistics, University of Athens School of Nursing, Athens, Greece; 3 Department of Clinical Therapeutics, “Alexandra” Hospital, University of Athens School of Medicine, Athens, Greece; 4 First Department of Medicine, “Laiko” General Hospital, University of Athens, Medical School, Athens, Greece; 5 Section of Biostatistics, Hellenic Cooperative Oncology Group, Data Office, Athens, Greece; 6 Second Department of Medical Oncology, “Metropolitan” Hospital, Piraeus, Greece; 7 Division of Oncology, Department of Medicine, University Hospital, University of Patras Medical School, Patras, Greece; 8 Department of Medical Oncology, University Hospital of Larissa, University of Thessaly School of Medicine, Larissa, Greece; 9 Department of Medical Oncology, “Hippokration” Hospital, Athens, Greece; 10 First Department of Surgery, “Papageorgiou” Hospital, Aristotle University of Thessaloniki School of Medicine, Thessaloniki, Greece; 11 Department of Obstetrics and Gynecology “Alexandra” Hospital, Athens, Greece; 12 Department of Cardiology, University of Ioannina Medical School, Ioannina, Greece; 13 Department of Medical Oncology, 424 Army General Hospital, Thessaloniki, Greece; 14 First Department of Medical Oncology, “Metropolitan” Hospital, Piraeus, Greece; 15 Second Department of Medical Oncology, “Hygeia” Hospital, Athens, Greece; 16 Third Department of Medical Oncology, “Agii Anargiri” Cancer Hospital, Athens, Greece; 17 Oncology Department, General Hospital of Chania, Crete, Greece; 18 Department of Medical Oncology, Ioannina University Hospital, Ioannina, Greece; 19 Oncology Section, Second Department of Internal Medicine, “Hippokration” Hospital, Athens, Greece

**Keywords:** Breast cancer, Dose-dense sequential chemotherapy, Anthracyclines, Taxanes, Trastuzumab

## Abstract

**Background:**

Dose-dense sequential chemotherapy including anthracyclines and taxanes has been established in the adjuvant setting of high-risk operable breast cancer. However, the preferable taxane and optimal schedule of administration in a dose-dense regimen have not been defined yet.

**Methods:**

From July 2005 to November 2008, 1001 patients (990 eligible) were randomized to receive, every 2 weeks, 3 cycles of epirubicin 110 mg/m^2^ followed by 3 cycles of paclitaxel 200 mg/m^2^ followed by 3 cycles of intensified CMF (Arm A; 333 patients), or 3 cycles of epirubicin followed by 3 cycles of CMF, as in Arm A, followed 3 weeks later by 9 weekly cycles of docetaxel 35 mg/m^2^ (Arm B; 331), or 9 weekly cycles of paclitaxel 80 mg/m^2^ (Arm C; 326). Trastuzumab was administered for one year to HER2-positive patients post-radiation.

**Results:**

At a median follow-up of 60.5 months, the 3-year disease-free survival (DFS) rate was 86%, 90% and 88%, for Arms A, B and C, respectively, while the 3-year overall survival (OS) rate was 96% in all arms. No differences were found in DFS or OS between the combined B and C Arms versus Arm A (DFS: HR = 0.81, 95% CI: 0.59-1.11, *P* = 0.20; OS: HR = 0.84, 95% CI: 0.55-1.30, *P* = 0.43). Among the 255 patients who received trastuzumab, 189 patients (74%) completed 1 year of treatment uneventfully. In all arms, the most frequently reported severe adverse events were neutropenia (30% vs. 27% vs. 26%) and leucopenia (12% vs. 13% vs. 12%), while febrile neutropenia occurred in fifty-one patients (6% vs. 4% vs. 5%). Patients in Arm A experienced more often severe pain (*P* = 0.002), neurological complications (*P* = 0.004) and allergic reactions (*P* = 0.004), while patients in Arm B suffered more often from severe skin reactions (*P* = 0.020).

**Conclusions:**

No significant differences in survival between the regimens were found in the present phase III trial. Taxane scheduling influenced the type of severe toxicities. HER2-positive patients demonstrated comparable 3-year DFS and OS rates with those reported in other similar studies.

**Trial registration:**

Australian New Zealand Clinical Trials Registry ACTRN12610000151033.

## Background

Breast cancer represents the most common cancer in women in western countries, while its incidence rate constantly increases in developing countries [1]. Adjuvant systemic therapy has significantly reduced the death rate of this disease [2]. In the last two decades, clinical research on adjuvant chemotherapy of early breast cancer (EBC) has been characterized by the conceptualization of new principles, such as dose-density and sequential chemotherapy [3] and the incorporation of taxanes to anthracycline-based chemotherapy.

The impact of dose-density (i.e., the increase of dose-intensity [DI] by reducing the interval between cycles with the use of granulocyte-colony stimulating factors [G-CSF]) on the outcome of patients treated with adjuvant chemotherapy has been extensively studied by several cooperative groups. A recently published meta-analysis showed that dose-dense adjuvant chemotherapy significantly improves disease-free survival (DFS) of patients with EBC compared to conventional chemotherapy without, however, demonstrating an apparent benefit in overall survival (OS) [4]. Further, sequential adjuvant chemotherapy significantly prolongs both DFS and OS over concurrent chemotherapy in this group of patients, as also shown in a meta-analysis including three trials with a total of over 8500 patients [5].

The impact of the incorporation of taxanes to adjuvant chemotherapy on the outcome of patients with EBC has been evaluated in numerous randomized trials. In an overview published recently by the Early Breast Cancer Trialists’ Cooperative Group [6], it was clearly shown that the addition of a taxane to anthracycline-based regimens, slightly, but significantly improved outcome.

Nevertheless, despite the proven beneficial effect of taxanes, the optimal taxane and the optimal schedule of administration remained for over a decade under intensive investigation. The weekly administration of docetaxel or paclitaxel has been studied in numerous clinical studies (reviewed in reference [7]) in patients with metastatic breast cancer. Further, several investigators incorporated docetaxel or paclitaxel weekly schedules to adjuvant chemotherapy regimens in large randomized trials in patients with EBC [8-12].

In 2005 and early 2006, the results of four randomized trials investigating the role of trastuzumab when added to adjuvant chemotherapy in patients with HER2-positive EBC were published [13-15]. These seminal trials demonstrated a remarkable reduction in relapse and death rates from the addition of trastuzumab [16,17]. The beneficial effect of trastuzumab was shown in two additional trials published a few years later, one in the adjuvant [18] and one in the neo-adjuvant setting [19].

The Hellenic Cooperative Oncology Group (HeCOG) has been involved in this field of clinical research by conducting two randomized trials exploring, in the first, the role of paclitaxel (Taxol®, Bristol Myers Squibb, Princeton, NJ) in a dose-dense sequential regimen with epirubicin and CMF [20] and in the second, the efficacy of a dose-dense sequential regimen with epirubicin, paclitaxel and CMF compared to that of concurrent administration of epirubicin and paclitaxel followed by CMF [21,22].

Following the completion of these studies, two feasibility studies were performed in the adjuvant setting, one with weekly docetaxel [23] and the other with weekly paclitaxel [24], sequentially administered after 3 cycles of epirubicin and 3 cycles of CMF given in a dose-dense fashion. Since the tolerability and safety of these regimens were satisfactory, we designed and conducted a 3-arm randomized trial (HE10/05) comparing the above-mentioned regimens with that of dose-dense epirubicin, paclitaxel and CMF (E-T-CMF). The latter was extensively studied in the two previously cited randomized trials [20-22] and served in the present trial as the control arm.

The primary endpoint of the trial was 3-year DFS. Secondary endpoints were 3-year OS and acute toxicity. Notably, the current trial incorporated a collateral translational research part, which included the prospective collection of biological material for the investigation of the predictive/prognostic significance of key biological markers and pathways. We report here the results of the first (interim) analysis of the HE10/05 trial at 5-year median follow-up.

## Methods

### Eligibility

Eligible women were older than 18 years with histologically confirmed node-positive (T_1-3_ N_1_ M_0_) or “intermediate risk” according to the 2005 St. Gallen criteria [25] (node negative patients with at least one of the following features: pT > 2 cm, or histological and/or nuclear grade 2-3, or presence of peritumoral vascular invasion, or HER2 gene overexpression and/or amplification, or age <35 years) adenocarcinoma of the breast. Patients had to have breast-conserving surgery with tumor-free margins or modified radical mastectomy, adequate hematologic, hepatic and renal function, performance status of 0 to 1 of the Eastern Cooperative Oncology Group (ECOG) scale, without evidence of significant cardiac disease (a normal left ventricular ejection fraction [LVEF] demonstrated by a Multiple Gated Acquisition [MUGA] scan or echocardiogram). Reasons for non-eligibility are described in detail [see Additional file 1].

Before randomization, each patient provided study specific written informed consent for participating in the trial and optionally a separate informed consent for providing biological material for research purposes. All clinical investigations related to the present study have been conducted according to the principles expressed in the Declaration of Helsinki.

The clinical protocol was approved by the Institutional Review Boards in participating centers (“Agii Anargiri” Cancer Hospital, “Alexandra” Hospital, “Attikon” University Hospital, “Errikos Dynan” Hospital, “Hygeia” Hospital, “Papageorgiou” Hospital, University Hospital of Ioannina, University Hospital of Larissa, University Hospital of Patras) and by the National Organization for Medicines. The trial was included in the Australian New Zealand Clinical Trials Registry (ANZCTR) and allocated the following Registration Number: ACTRN12610000151033.

Pretreatment evaluation included medical history, clinical examination, chest X-rays and abdominal ultrasonography (or computed tomography [CT] scans in patients with more than nine infiltrated axillary nodes or if clinically indicated), bone scans, ejection fraction (EF), complete blood count (CBC) and a comprehensive biochemistry panel. CBC and biochemistries were repeated before each cycle and EF after the completion of chemotherapy and then every 4 months during treatment with trastuzumab. Furthermore, CBC was done between cycles in the case of fever over 38^o^C, severe stomatitis or diarrhea.

### Treatment

Stratified block randomization (1:1:1), balanced by center, was performed centrally at the HeCOG Data Office in Athens by telephone. Stratification factors included menopausal status (pre vs. post), hormonal receptor status (positive vs. negative) and number of involved axillary lymph nodes (0 vs. 1-3 vs. ≥4). Patients were randomized to receive one of the following three chemotherapeutic schedules: three cycles of epirubicin (E, 110 mg/m^2^) every 2 weeks followed by 3 cycles of paclitaxel (T, 200 mg/m^2^) every 2 weeks followed by 3 cycles of intensified CMF (cyclophosphamide 840 mg/m^2^, methotrexate 57 mg/m^2^ and fluorouracil 840 mg/m^2^) every 2 weeks (Arm A, E-T-CMF), or three cycles of epirubicin followed by 3 cycles of CMF, as in Arm A, followed 3 weeks later by 9 consecutive weekly cycles of docetaxel (wD) 35 mg/m^2^ (Arm B, E-CMF-wD), or 9 consecutive weekly cycles of paclitaxel (wT) 80 mg/m^2^ (Arm C, E-CMF-wT).

G-CSF was given following each cycle in Arm A and during the intensified phase of epirubicin and CMF treatments in Arms B and C. Dose modifications are shown in detail [see Additional file 1]. The National Cancer Institute Common Terminology Criteria for Adverse Events, Version 3.0 were used to assess toxicity. Patients with HER2-positive tumors were treated with trastuzumab, initially at a dose of 8 mg/kg as a loading dose, and subsequently 6 mg/kg every three weeks for one year. Initially, HER2-positive tumors were considered those with an immunohistochemistry (IHC) score of 3+ (uniform, intense membrane staining of >10% of invasive tumor cells), a fluorescence in situ hybridization (FISH) result of ≥6 HER2 gene copies, or a FISH ratio (HER2 gene signals to chromosome 17 signals) of >2.0. Following the 2007 publication of the American Society of Clinical Oncology/College of American Pathologists guideline recommendations for HER2 testing in breast cancer [26], the criteria for characterizing a tumor as HER2-positive were updated (the FISH ratio was changed to >2.2).

Ondansetron ± dexamethasone were recommended as antiemetic treatment in all patients. Radiation therapy (RT) was required for all patients who underwent partial mastectomy or those with tumor size ≥5 cm and/or more than 4 positive lymph nodes, irrespective of the type of surgery (conservative or radical). Details for the RT technique are given [see Additional file 1]. RT was initiated 3-4 weeks following the completion of chemotherapy.

Premenopausal patients with hormone receptor-positive status received oral tamoxifen 20 mg daily for 5 years and goserelin 10.8 mg subcutaneously every 3 months for 2 years. Postmenopausal patients with hormone receptor-positive status were treated daily with anastrazole 1 mg orally for 5 years. Postmenopausal patients were considered those without menses for the last two years or those older than 50 years who underwent a hysterectomy for non-malignant reasons. Tumors were considered hormone receptor-positive if ≥1% of tumor cell nuclei were stained. It has to be noted that in the present analysis, hormone receptor status and HER2 status are presented as assessed by local laboratories. Trastuzumab and hormonal therapies were administered following the completion of chemotherapy and RT.

Data entry was performed in a central database by trained HeCOG data managers located at the different participating centers. The study was internally monitored by certified HeCOG personnel.

### Follow-up

All patients were followed at the Clinic, at study entry, every six months for the first five years and annually thereafter with clinical examinations, CBC, biochemistry panels, serological markers, chest X-rays and abdominal ultrasonography (or CT scans if clinically indicated). Mammography and ultrasonography of the breasts were performed annually. Bone scans were not routinely done after the third year, except when clinically indicated.

### Statistical analysis

In this multicenter phase III randomized, open-label, comparative trial (parallel assignment and efficacy study) the primary objective on an intent-to-treat analysis was DFS. Based on the initial hypothesis that the epirubicin, CMF and weekly docetaxel or weekly paclitaxel arms (Arms B and C) were equally effective on DFS, a comparison of the combined Arms B and C to the epirubicin, paclitaxel and CMF arm (Arm A), was of interest. One thousand patients were required to be randomized to the study to detect a 5% difference between the combined arms (Arms B and C) vs. the control arm (Arm A), with a two-sided test at the 5% level of significance and a power of 80%, assuming a 3-year DFS rate of 80% for the control arm. The study accrual rate was estimated at 330 patients per year and the maximum study duration was estimated to be 8.1 years for observing a total of 329 relapses. An interim analysis based on the O’Brien Fleming boundary values was to be performed when 50% of the events had been reached.

DFS was defined based on the interval from study entry to first locoregional recurrence, first distant metastasis, contralateral breast cancer, secondary neoplasm, death from the disease or death from any cause, whichever occurred first. OS was measured from study entry until death from any cause. Surviving patients were censored at the date of last contact.

Fisher’s exact or Pearson chi-square tests were used for group comparisons of categorical data, while for continuous data the non-parametric Mann-Whitney or the Kruskall-Wallis tests were used, where appropriate. Survival distributions were estimated using the Kaplan-Meier method. The significance nominal level for the tests of the hypotheses was set at p < 0.05.

For the univariate and multivariate analyses, Cox proportional hazards models were used. In the multivariate setting, model choice was performed using backward selection criteria with *P* < 0.10, including in the initial step clinicopathological parameters, menopausal status (post vs. premenopausal), number of positive nodes (≥4 and 1-3 vs. 0), tumor size (>2 vs. ≤2), histological grade (3 vs. 1 + 2), ER/PgR status (positive vs. negative) and HER2 status (positive vs. negative), in the presence of randomization arm (combined Arms B and C vs. Arm A). The final multivariate models are presented by forest plots.

All endpoints except adverse events and treatment characteristics were analyzed according to the intent-to-treat (ITT) principle. The reported *P*-values are two-sided. Survival status was updated in July 2012. The SPSS (version 15.0, IBM Corporation, Armonk, NY) and SAS (version 9.3, SAS Institute Inc., Cary, NC) software were used for statistical analysis.

## Results

### Patient population

From July 2005 until November 2008, 1001 patients were randomized (990 eligible; 333, 331 and 326 in Arms A, B and C, respectively). Eleven patients were deemed non- eligible (4 patients with M1 disease, one with bilateral breast cancer, one with co-existing renal cancer, one with inadequate examination of lymph nodes and 4 with violations in the randomization procedure). Furthermore, 4 patients withdrew consent prior to receiving protocol treatment.

**Figure 1:**
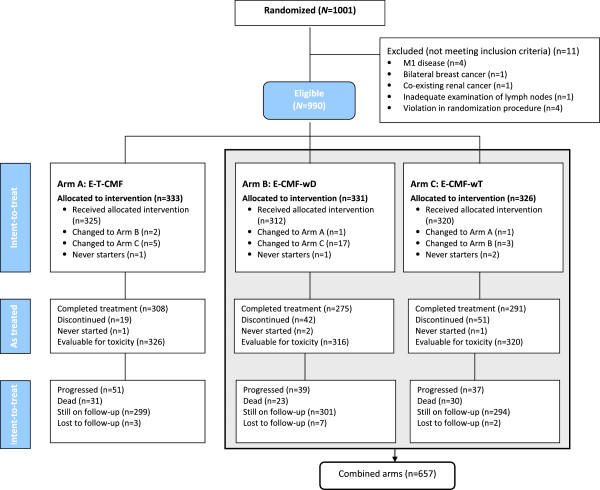
CONSORT diagram.

The progress of patients through the various stages of the trial according to the Consolidation Standards of Reporting Trials (CONSORT) flow diagram is shown in Figure 1. Selected patient and tumor characteristics are presented in Table 1. All characteristics were well balanced between the treatment arms (Pearson chi-square test, all *P*-values above 0.05).

**Table 1:** **Patient and tumor characteristics per study arm**^1^**and in the total study population**

	**Arm A: E-T-CMF N = 333**	**Arm B: E-CMF-wD N = 331**	**Arm C: E-CMF-wT N = 326**	**Total study population N = 990**
Age				
Median (range)	53 (28-79)	53 (21-78)	54 (23-78)	53 (21-79)
	***N*** **(%)**	***N*** **(%)**	***N*** **(%)**	***N*** **(%)**
Menopausal status				
Premenopausal	155 (46.5)	157 (47.4)	149 (45.7)	461 (46.6)
Postmenopausal	178 (53.5)	174 (52.6)	177 (54.3)	529 (53.4)
Surgery				
MRM	167 (50.2)	163 (49.2)	175 (53.7)	505 (51.0)
Partial mastectomy	166 (49.8)	168 (50.8)	151 (46.3)	485 (49.0)
Tumor size				
≤2	159 (47.7)	139 (42.0)	119 (36.5)	417 (42.1)
2.1-5	155 (46.5)	169 (51.1)	183 (56.1)	507 (51.2)
>5	19 (5.7)	23 (6.9)	24 (7.4)	66 (6.7)
Positive nodes				
0	83 (24.9)	80 (24.2)	83 (25.5)	246 (24.8)
1-3	136 (40.8)	136 (41.1)	136 (41.7)	408 (41.2)
≥4	114 (34.2)	115 (34.7)	107 (32.8)	336 (33.9)
Histological grade				
1	25 (7.5)	16 (4.8)	20 (6.1)	61 (6.2)
2	155 (46.5)	141 (42.6)	152 (46.6)	448 (45.3)
3	153 (45.9)	174 (52.6)	154 (47.2)	481 (48.6)
Histological type				
Invasive ductal NOS	274 (82.3)	273 (82.5)	279 (85.6)	826 (83.4)
Invasive lobular	31 (9.3)	28 (8.5)	22 (6.7)	81 (8.2)
Mixed type	18 (5.4)	18 (5.4)	11 (3.4)	47 (4.7)
Medullary	6 (1.8)	6 (1.8)	4 (1.2)	16 (1.6)
Mucinous	2 (0.6)	1 (0.3)	2 (0.6)	5 (0.5)
Papillary	1 (0.3)	3 (0.9)	3 (0.9)	7 (0.7)
Tubular	-	-	1 (0.3)	1 (0.1)
Apocrine	-	1 (0.3)	1 (0.3)	2 (0.2)
Metaplastic	-	1 (0.3)	-	1 (0.1)
Neuroendocrine	-	-	1 (0.3)	1 (0.1)
Myeloepithelioma	-	-	1 (0.3)	1 (0.1)
Other	1 (0.3)	-	1 (0.3)	2 (0.2)
Hormone receptor status				
Negative	74 (22.2)	74 (22.4)	71 (21.8)	219 (22.1)
Positive	259 (77.8)	257 (77.6)	255 (78.2)	771 (77.9)
HER2 overexpression				
No	239 (71.8)	240 (72.5)	237 (72.7)	716 (72.3)
Yes	94 (28.2)	91 (27.5)	89 (27.3)	274 (27.7)
Triple-negative				
No	292 (87.7)	288 (87.0)	291 (89.3)	871 (88.0)
Yes	41 (12.3)	43 (13.0)	35 (10.7)	119 (12.0)
Post-chemotherapy treatment				
Adjuvant hormonal therapy				
No	83 (24.9)	85 (25.7)	83 (25.5)	251 (25.4)
Yes	250 (74.1)	246 (74.3)	243 (74.5)	739 (74.6)
Tamoxifen	122 (36.6)	113 (34.1)	105 (32.2)	340 (34.3)
LH-RH	101 (30.3)	94 (28.4)	97 (29.8)	292 (29.5)
Aromatase inhibitor	150 (45.0)	152 (45.9)	163 (50.0)	465 (47.0)
Fulvestrant	1 (0.3)	1 (0.3)	1 (0.3)	3 (0.3)
Adjuvant radiotherapy				
No	89 (26.7)	95 (28.7)	89 (27.3)	273 (27.6)
Yes	244 (73.3)	236 (71.3)	237 (72.7)	717 (72.4)
Trastuzumab treatment				
No	243 (73.0)	247 (74.6)	245 (75.2)	735 (74.2)
Yes	90 (27.0)	84 (25.4)	81 (24.8)	255 (25.8)

^**1**^No significant differences between treatment arms were found (Pearson chi-square test).

*N* number of patients, *MRM* modified radical mastectomy, *NOS* not otherwise specified.

### Treatment compliance

Totally, 885 (89.4%) patients (306 in Arm A, 279 in Arm B and 300 in Arm C) completed chemotherapy. Dose intensities (DI) of all drugs are given in Table 2. The discontinuation rate was significantly lower in the E-T-CMF arm [6.7% in Arm A vs. 12.5% in Arms B and C (12.3% and 12.8%, respectively), *P* = 0.004]. Treatment compliance and reasons for early chemotherapy discontinuation are summarized [see Additional file 2: Table S1]. The main reasons for discontinuation, observed in 105 patients (10.6% of the total study population), were toxicity in 38 of the 105 patients (36%) and voluntary withdrawal in 38 patients (36%).

**Table 2:** Treatment characteristics (as treated population)

	**Arm A: E-T-CMF N = 327**	**Arm B: E-CMF-wD N = 317**	**Arm C: E-CMF-wT N = 342**
	**N (%)**	**N (%)**	**N (%)**
**Number of cycles per patient**			
1	1 (0.3)	2 (0.6)	3 (0.9)
2	2 (0.6)	2 (0.6)	3 (0.9)
3	1 (0.3)	2 (0.6)	-
4	3 (0.9)	2 (0.6)	3 (0.9)
5	4 (1.2)	-	-
6	3 (0.9)	2 (0.6)	6 (1.8)
7	1 (0.3)	1 (0.3)	1 (0.3)
8	6 (1.8)	2 (0.6)	2 (0.6)
9	306 (93.6)	2 (0.6)	4 (1.2)
10	-	2 (0.6)	-
11	-	1 (0.3)	-
12	-	3 (0.9)	2 (0.6)
13	-	4 (1.3)	7 (2.0)
14	-	12 (3.8)	10 (2.9)
15	-	280 (88.3)	301 (88.0)
**Total cycles given**	2867	4560	4886
Median (range)	9 (1-9)	15 (1-15)	15 (1-15)
	**Median (range)**	**Median (range)**	**Median (range)**
**Dose intensity (DI)**			
Epirubicin	54 (26-58)	54 (26-64)	54 (30-64)
Paclitaxel	100 (57-105)	-	78 (20-121)
Cyclophosphamie	410 (204-650)	414 (191.5-490)	410 (170-486)
Methotrexate	28 (14-44)	28 (13.2-43)	28 (11-43)
Fluorouracil	410 (204-650)	414 (191.5-490)	410 (170-486)
Docetaxel	-	34 (15.7-80)	-
**Relative dose intensity (RDI)**			
Epirubicin	1.0 (0.5-1.1)	1.0 (0.5-1.2)	1.0 (0.6-1.2)
Paclitaxel	1.0 (0.6-1.1)	-	1.0 (0.3-1.5)
Cyclophsphamide	1.0 (0.5-1.6)	1.0 (0.5-1.2)	1.0 (0.4-1.2)
Methotrexate	1.0 (0.5-1.5)	1.0 (0.5-1.5)	1.0 (0.4-1.5)
Fluorouracil	1.0 (0.5-1.6)	1.0 (0.5-1.2)	1.0 (0.4-1.2)
Docetaxel	-	1.0 (0.5-2.3)	-

*N* number of patients.

Among 274 patients with HER2-positive tumors, trastuzumab was administered in 254 patients (90, 84 and 80 in Arms A, B and C, respectively). Twenty patients (7.3%) did not receive trastuzumab, despite being found with HER2-positive tumors (12 patients because of voluntary withdrawal, 2 never starters and 6 with early relapse). One HER2-negative patient (IHC score 2+), randomized to Arm C, was treated with trastuzumab for three months, until the FISH result was reported to be negative. Compliance of patients to treatment with trastuzumab is presented in Table 3. Among those who received trastuzumab, 189 patients (74%) (69, 58 and 62) completed 1 year of treatment uneventfully. Reasons for trastuzumab discontinuation are shown in the footnote of Table 3. Finally, there were 13 patients [7 in Arm A vs. 6 in Arms B and C, *P* = 0.23] who were treated with trastuzumab for more than 1 year, based on patient preference.

**Table 3:** Treatment compliance to trastuzumab

	**Arm A: E-T-CMF N = 333 N (%)**	**Arm B: E-CMF-wD N = 331 N (%)**	**Arm C: E-CMF-wT N = 326 N (%)**	**Total study population N = 990 N (%)**
Received trastuzumab				
No	243 (73)	247 (75)	245 (75)	735 (74)
Yes	90 (27)	84 (25)	81 (25)	255 (26)
Completed 1 year uneventfully	69 (77)	58 (69)	62 (77)	189 (74)
Discontinued				
Temporarily^1^	13 (14)	16 (19)	12 (15)	41 (16)
Permanently^2^	8 (9)	10 (12)	7 (9)	25 (10)

^1^Treatment delay (*n* = 5), asymptomatic reduction of ejection fraction (*n* = 1), infection (*n* = 3), voluntary withdrawal (*n* = 28), not defined (*n* = 4).

The temporary discontinuation of the trastuzumab treatment was short in duration, with a median discontinuation time of 3 weeks, with 39 of the 41 patients (95%) eventually receiving a full year of trastuzumab treatment.

^2^Chronic heart failure (*n* = 3), asymptomatic reduction of ejection fraction (*n* = 4), disease progression (*n* = 7), withdrawal of consent (*n* = 9), other (*n* = 2).

*N* number of patients.

### Efficacy

**Figure 2:**
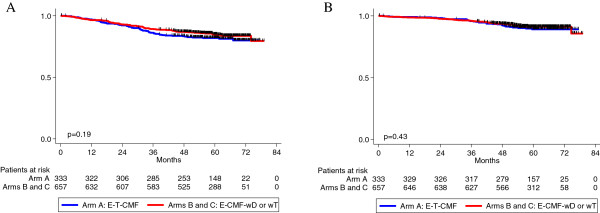
**Disease-free survival (A) and overall survival (B) in the total study population.** Patients treated in Arms B (E-CMF-wD) and C (E-CMF-wT) were combined and compared to the patients treated in Arm A (E-T-CMF). Log-rank p-values are reported.

After a median follow-up time of 60.5 months (range, 0.1-79.0), 160 disease-defining events (61 vs. 50 and 49) were recorded. At the time of this analysis (July 2012), 129 (13%) of the patients (51 vs. 40 and 38) had demonstrated disease progression and 88 (8.9%) (33 vs. 25 and 30) had died. Sites of relapse according to randomization arm are presented in detail [see Additional file 2: Table S2]. Seven patients (0.7%) developed second neoplasm (colorectal cancer in 2, contralateral breast cancer in 2, lung cancer, ovarian cancer and peritoneal carcinomatosis in one patient each). One additional patient in Arm B was diagnosed, 3 years after the completion of chemotherapy, with secondary acute myelogenous leukemia. The majority of the patients (69%) died from tumor disease, while seven patients (0.7%) died during adjuvant chemotherapy from causes displayed in Table 4. Three-year DFS rates were 86.1%, 90.3% and 88.3% in arms A, B and C, respectively, while 3-year OS rates were 95.8%, 96.3% and 95.7%. No significant differences were observed in DFS and OS between the combined B and C Arms versus Arm A (DFS: Hazard ratio [HR] = 0.81, 95% Confidence Interval [CI]: 0.59-1.11, Wald’s *P* = 0.20; OS: HR = 0.84, 95% CI: 0.55-1.30, Wald’s *P* = 0.43) (Figure 2). Moreover, Arms B and C were equally effective on DFS and OS, as initially assumed [see Additional file 2: Figure S1].

**Table 4:** Cause of death during chemotherapy

**Cause of death**	**N**	**Treatment arm**	**Time from initiation of CT until death (weeks)**
Febrile neutropenia	1	B	9
Febrile neutropenia	1	A	19
Infection (Hepatitis B reactivation)	1	A	10
Pulmonary embolism	1	C	13
Acute myocardial infarction	1	C	3
Acute respiratory failure	1	B	15
Unspecified	1	C	18

*N* number of patients, *CT* chemotherapy.

**Figure 3:**
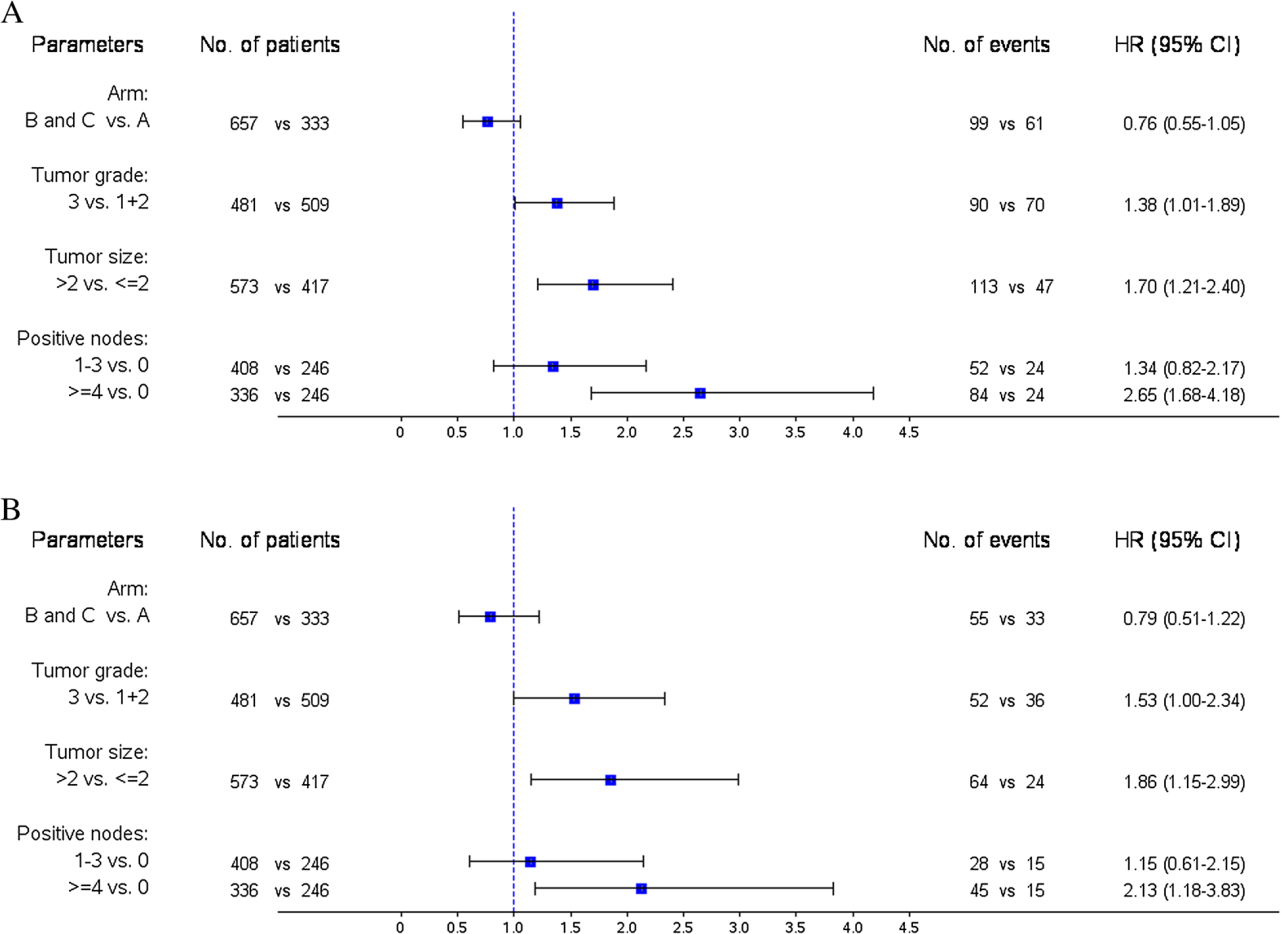
Multivariate Cox regression analysis for DFS (A) and OS (B) presented by forest plots.

The present interim analysis was conducted at approximately half of the events. Follow-up is ongoing until the required events for the primary endpoint are observed. The observed event rate however, is approximately half of what was expected and thus, it will take much longer than the anticipated study duration to observe the 329 DFS events.Multivariate Cox regression analysis results for DFS and OS are shown in Figure 3. Tumor grade, tumor size and number of positive lymph nodes were identified as independent prognostic factors for both DFS and OS.

**Figure 4:**
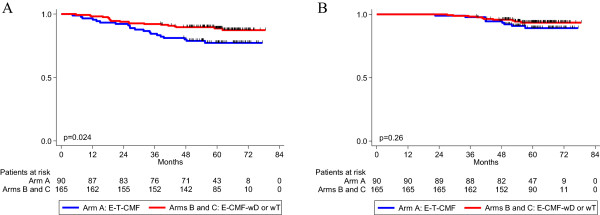
**Disease-free survival (A) and overall survival (B) in patients treated with trastuzumab.** Patients treated in Arms B (E-CMF-wD) and C (E-CMF-wT) were combined and compared to the patients treated in Arm A (E-T-CMF). Log-rank p-values are reported.

In an exploratory analysis only among patients receiving trastuzumab, those treated with weekly taxanes had significantly longer DFS (*P* = 0.024, log-rank) than those in the control arm; OS however was similar (*P* = 0.26) (Figure 4).

### Toxicity

The incidence of severe adverse events is shown in Table 5. The most common were neutropenia (28.0%), leukopenia (12.4%), febrile neutropenia (5.3%), metabolic disturbances (4.3%), mucositis (3.5%) and infection (3.1%). Patients in Arm A more often experienced severe arthralgias/myalias (*P* = 0.002), neurological complications (*p* = 0.004) and allergic reactions (*P* = 0.004), while patients in Arm B more often suffered from severe skin reactions (*P* = 0.020). Febrile neutropenia occurred in 51 patients despite the use of prophylactic G-CSF and was fatal in two patients, one in Arm A and one in Arm B. Adverse events of any grade, per treatment arm, are shown in detail [see Additional file 2: Table S3].

**Table 5:** Incidence of severe adverse events according to treatment arm (as treated)

	**Arm A: E-T-CMF**						**Arm B: E-CMF-wD**						**Arm C: E-CMF-wT**					
	**N = 326**						**N = 316**						**N = 320**					
	**Grade III**		**Grade IV**		**Grade V**		**Grade III**		**Grade IV**		**Grade V**		**Grade III**		**Grade IV**		**Grade V**	
	**N**	**(%)**	**N**	**(%)**	**N**	**(%)**	**N**	**(%)**	**N**	**(%)**	**N**	**(%)**	**N**	**(%)**	**N**	**(%)**	**N**	**(%)**
Hemoglobin	6	1.8					3	0.9					6	1.9				
Leucocytes	28	8.6	11	3.4			36	11.4	5	1.6			32	10.0	7	2.2		
Lymphopenia	2	0.6					4	1.3					1	0.3				
Neutrophils	55	16.9	44	13.5			61	19.3	24	7.6			49	15.3	36	11.3		
Platelets	1	0.3	1	0.3			1	0.3	1	0.3			3	0.9	1	0.3		
Febrile neutropenia	17	5.2	2	0.6	1	0.3	12	3.8	1	0.3	1	0.3	13	4.1	4	1.3		
Gastrointestinal							4	1.3					4	1.3				
Metabolic/laboratory	12	3.7					7	2.2	1	0.3			16	5.0	5	1.6		
Dermatology/skin^1^	5	1.5					9	2.8					1	0.3				
Pain^2^	17	5.2					3	0.9					5	1.6				
Pulmonary/Upper respiratory			2	0.6			2	0.6	1	0.3	1	0.3	1	0.3				
Constitutional							1	0.3					1	0.3				
Fatigue	4	1.2					7	2.2					9	2.8	1	0.3		
Diarrhea	3	0.9					8	2.5					3	0.9				
Nausea	7	2.1					6	1.9					10	3.1				
Neurology^3^	16	4.9					4	1.3					4	1.3				
Vomiting	5	1.5					5	1.6	1	0.3			4	1.3				
Mucositis	6	1.8	1	0.3			17	5.4					10	3.1				
Infection	7	2.1			1	0.3	15	4.7					7	2.2				
Occular							2	0.6										
Allergy^4^	8	2.5													2	0.6		
Edema							1	0.3										
Vascular	2	0.6					3	0.9					7	2.2			1	0.3
Cardiac			1	0.3			2	0.6					1	0.3			1	0.3
Anorexia							1	0.3										

^1^*P* = 0.020 (Fisher’s test); ^2^*P* = 0.002; ^3^*P* = 0.004; ^4^*P* = 0.004.

*N* number of patients.

## Discussion

A few years ago, a real breakthrough occurred in the management of patients with EBC and HER2-positive disease, with the publication of four randomized trials investigating the addition of trastuzumab to adjuvant chemotherapy [13-15]. The primary endpoint in all trials was DFS. Trastuzumab was administered concurrently or sequentially to a variety of chemotherapeutic regimens for at least 1 year. The only trial testing a shorter duration was the Finnish trial [15], in which trastuzumab was given for 9 weeks. The HERA trial [14] randomized patients to receive trastuzumab for 1 or 2 years. In the following years, the results of two additional trials [18,27] and of further analyses with longer follow-up of the four initial trials were also published [28-31]. In all studies, except one [27], DFS (and OS in some) was significantly improved with the addition of trastuzumab [16,17]. It is notable that, in the final analysis of the FinHER trial [31] with a median follow up of 5 years, even though the beneficial effect of trastuzumab on DFS was not present, a preplanned exploratory analysis within the HER2-positive group revealed that the subset of patients treated with docetaxel, trastuzumab and FEC had a superior DFS to that of patients who received docetaxel and FEC (HR = 0.32; *P* = 0.023) and to that of patients treated with vinorelbine, FEC and trastuzumab (HR = 0.31; *P* = 0.020). No significant difference was observed in OS.

In the present study, we hypothesized that modifying the schedule of administration of taxanes i.e., docetaxel or paclitaxel to weekly instead of 2-weekly in an adjuvant dose-dense regimen, might improve DFS in patients with intermediate or high-risk operable breast cancer. In the current analysis, a significant difference in DFS between the treatment regimens has not been detected. The conditional power at half of the information time is 44% and the study continues to completion.

The optimal schedule of both taxanes following anthracycline-based chemotherapy was investigated in an Intergroup trial lead by Eastern Cooperative Oncology Group (ECOG) [11]. In that pivotal trial, 4950 women with node-positive or high-risk node-negative breast cancer were randomized to receive postoperatively four cycles of doxorubicin and cyclophosphamide (AC) every three weeks followed by docetaxel or paclitaxel at 3-week intervals for four cycles or at 1-week intervals for 12 cycles. Weekly paclitaxel following anthracycline/cyclophosphamide chemotherapy appeared to be more effective than 3-weekly paclitaxel (HR = 1.27, *P* = 0.006 and HR = 1.32, *P* = 0.01 for DFS and OS, respectively). Conversely, 3-weekly docetaxel was superior to 3-weekly paclitaxel in DFS (HR = 1.23, *P* = 0.02), although not in OS (HR = 1.13, *P* = 0.25). At the time we designed the present study, the results of the ECOG E1199 trial [11] were not available, and thus assuming similar efficacy, we used in the experimental arms the weekly schedules of both taxanes.

A notable difference among the two trials was that our patients with HER2-positive tumors received trastuzumab for 1 year. Given the available information at the time of our study design, we selected to offer trastuzumab for 1 year sequentially to chemotherapy, since this strategy was in accordance to that adopted in two of the published adjuvant trastuzumab studies [13,14]. Importantly, 3-year and 5-year DFS rates observed in the present analysis were similar to those reported in the pivotal adjuvant trastuzumab trials.

Nevertheless, despite the wealth of clinical data available on the adjuvant treatment with trastuzumab, critical issues, such as optimal duration (1 year or shorter duration), sequence (concurrently or sequentially to chemotherapy), optimal chemotherapeutic regimen or schedule of administration of trastuzumab, are still a matter of controversy. Regarding the issue of optimal duration of treatment with trastuzumab, information on head to head comparisons between 1 year and shorter duration (6 months) [32] or longer duration (2 years) [33] have recently been reported, suggesting that at present 1 year of adjuvant trastuzumab should remain the standard treatment. Results from the Finish Synergism or Long Duration (SOLD; NCT00593697) study, exploring the tantalizing issue of testing 9 weeks versus 1 year of trastuzumab, which is tightly associated with patients’ convenience and reduced toxicity and treatment costs, are still pending.

As far as the issue of sequence is concerned, data from the recent analysis of the N9831 trial [34] and a meta-analysis [35] strongly support the superiority of concurrent over the sequential use of trastuzumab.

It is generally accepted that the type of chemotherapy given concurrently with trastuzumab does not affect efficacy. Even though this is probably true in the management of metastatic breast cancer, it might not hold true in the adjuvant setting. Data from the FinHER trial [31] indicate that the drugs or type of chemotherapy delivered concurrently with trastuzumab probably matter. More information from prospectively designed studies is needed to shed light on this issue.

In three of the five published adjuvant trastuzumab trials [14,18,27], trastuzumab was given on a weekly basis. Even though this schedule is widely used in patients with metastatic breast cancer [36,37], experience with its use in the adjuvant setting is limited. Whether efficacy of 3-weekly trastuzumab, as given in the present study, is comparable to that of weekly in the adjuvant setting of EBC, an issue that is assumed but not proven, is not known. It is expected that a number of ongoing trials using the 3-weekly schedule will increase our knowledge on this critical issue, also associated with convenience and reduced cost.

The toxicity profile of chemotherapy in the present study was similar to that reported in our previously conducted randomized trials [20-22], even though the dose of paclitaxel in the control arm was reduced by 20%. Patients randomized to Arm A developed more frequently severe allergic reactions, pain and peripheral neuropathy, while patients in Arm B suffered more frequently from severe skin reactions. Despite the prophylactic use of G-CSF, 5% of patients developed febrile neutropenia, which was fatal in two cases. Furthermore, among 255 patients that were treated with trastuzumab, only 189 (74%) completed 1 year of treatment uneventfully.

The discontinuation rate of 1-year trastuzumab therapy ranged from 25% to 31% in most adjuvant trials [13,27], remarkably similar to the 26% recorded in our trial. It should be noted however, that our study utilized a sequential chemotherapy/trastuzumab design, as opposed to the two referenced trials that utilized a concurrent chemotherapy/trastuzumab design, in which patients received trastuzumab immediately after the completion of the anthracycline and upon initiation of the taxane treatment. Of note, in the HERA trial [14] this rate was 8.5% excluding, however, those patients who discontinued trastuzumab because of disease relapse. The higher discontinuation rate observed in our study might also be due to the fact that our study is, to our knowledge, the only trial that used trastuzumab in a dose-dense adjuvant chemotherapy design. The observed relatively high discontinuation rate of both the chemotherapy and trastuzumab regimens, mainly due to toxicity, constitute a limitation of our study together with the small, however non-negligible number of patients that changed arm during treatment.

## Conclusions

In conclusion, the present trial continues to investigate, whether a 5% difference exists in 3-year DFS between the weekly taxane regimens and the control arm. DFS rates in patients with HER2-positive tumors were similar to those reported in the seminal adjuvant trastuzumab trials. In a subgroup analysis of trastuzumab-treated patients only, DFS was significantly longer among patients receiving a weekly taxane. Nevertheless, this was an unplanned analysis and therefore, these data should merely be considered as hypothesis generating, at present. Importantly, to the best of our knowledge, this is the first randomized trial, which clearly showed that the administration of trastuzumab for 1 year following adjuvant dose-dense chemotherapy is feasible and safe.

## Abbreviations

AC: Doxorubicin/cyclophosphamide; ANZCTR: Australian New Zealand Clinical Trials Registry; CBC: Complete blood count; CI: Confidence interval; CMF: Cyclophosphamide, methotrexate, fluorouracil; CT: Computed tomography; DFS: Disease-free survival; DI: Dose-intensity; E: Epirubicin; EBC: Early breast cancer; ECOG: Eastern Cooperative Oncology Group; EF: Ejection fraction; ER: Estrogen receptors; FEC: Fluorouracil, epirubicin, cyclophosphamide; FISH: Fluorescence in situ hybridization; HECOG: Hellenic Cooperative Oncology Group; HER: Human epidermal growth factor receptor; HR: Hazard ratio; IHC: Immunohistochemistry; ITT: Intent to treat; LVEF: Left ventricular ejection fraction; MUGA: Multiple gated acquisition; OS: Overall survival; PgR: Progesterone receptors; RT: Radiation therapy; T: Paclitaxel; vs.: Versus; wD: Weekly docetaxel; wT: Weekly paclitaxel.

## Competing interests

The senior investigator (GF) has received Commercial Research Funding by Roche Hellas SA and Genesis Pharma SA, Athens, Greece. The rest of the authors declare that they have no competing interests.

## Authors’ contributions

GF conceived of the study, participated in its design and coordination, contributed to the acquisition, analysis and interpretation of data and drafted the manuscript. UD conceived of the study, participated in its design, contributed to the analysis and interpretation of data and drafted the manuscript. CP conceived of the study, participated in its design, contributed to the acquisition of data and helped to draft the manuscript. ET contributed to the acquisition of data and helped to draft the manuscript. HG conceived of the study, participated in its design, contributed to the acquisition of data and helped to draft the manuscript. AGE participated in the analysis and interpretation of data and drafted the manuscript. IX contributed to the acquisition of data. CC conceived of the study, participated in its design and contributed to the acquisition, analysis and interpretation of data. AK conceived of the study, participated in its design and contributed to the acquisition, analysis and interpretation of data. CNP contributed to the acquisition of data. PP contributed to the acquisition of data. SM contributed to the acquisition of data. CM contributed to the acquisition of data. CD contributed to the acquisition of data. PK contributed to the interpretation of data. CK contributed to the acquisition of data. DB conceived of the study, participated in its design and contributed to the acquisition of data. PK conceived of the study, participated in its design and contributed to the acquisition of data. ES conceived of the study, participated in its design and contributed to the acquisition of data. IV contributed to the acquisition of data. NP conceived of the study, participated in its design, contributed to the acquisition of data and helped to draft the manuscript. DP conceived of the study, participated in its design, contributed to the acquisition, analysis and interpretation of data and helped to draft the manuscript. MAD conceived of the study, participated in its design, contributed to the acquisition, analysis and interpretation of data and drafted the manuscript. All authors read and approved the final manuscript.

## Pre-publication history

The pre-publication history for this paper can be accessed here:

http://www.biomedcentral.com/1471-2407/14/515/prepub

## Supplementary Material

Additional file 1 **Exclusion criteria, dose modification and radiation therapy details.** Click here for file

Additional file 2: Table S1 Treatment compliance and discontinuation reasons. **Table S2.** Sites of relapse. **Table S3.** Incidence of adverse events according to treatment arm (as treated). **Figure S1.** Disease-free survival (A) and overall survival (B) of patients treated in Arm A (E-T-CMF), Arm B (E-CMF-wD) and Arm C (E-CMF-wT). Log-rank p-values are reported. Click here for file
