# Supplementary material for: Dose-dense sequential adjuvant chemotherapy followed, as indicated, by trastuzumab for one year in patients with early breast cancer: first report at 5-year median follow-up of a Hellenic Cooperative Oncology Group randomized phase III trial
Source: BMC Cancer. 2014 Jul 15;14:515. doi: 10.1186/1471-2407-14-515 (PMC4223601; doi:10.1186/1471-2407-14-515)
Supplement: Additional file 1 — Exclusion criteria, dose modification and radiation therapy details. [file 1471-2407-14-515-S1.doc]

#### EXCLUSION CRITERIA

- History of myocardial infarction within the previous 12 months or heart failure (including cardiac insufficiency controlled by digitalis and diuretics) or arrhythmias requiring medication or uncontrolled arterial hypertension (blood pressure >200/110 mm Hg).
- Documented residual or metastatic disease.
- Prior chemotherapy, hormonal or radiation therapy.
- Pregnant or in puerperium period women, or patients unwilling to follow adequate contraceptive methods during the treatment period.
- History of prior cancer except for curatively treated basal-cell carcinoma of the skin or in situ carcinoma of the cervix of the uterus.
- Patients who cannot fully understand and complete the inform consent form, or patients who can not follow treatment or follow-up schedule.

#### DOSE MODIFICATIONS

In case of grade 3 or 4 hematological or non-hematological toxicity (except for alopecia, nausea and vomiting), the dose of drugs will be reduced in all subsequent cycles by 25% or 50%, respectively.

**Hematological recovery** (value at day of scheduled retreatment, i.e. day 14)

The ANC count must be >1.5 x 10^9^/L and the platelet count >100 x 10^9^/L prior to the beginning of the following course of treatment.

For patients who do not achieve hematological recovery on day 21, CBC should be done every week until ANC>1.5x10^9^/L and platelet count >100 x 10^9^/L. If hematological recovery is achieved (before day 28), treatment is administered immediately.

If hematological recovery is not achieved on day 28 of the cycle (or on day 21 during docetaxel treatment), the patient will be taken off treatment.

During treatment with weekly docetaxel, chemotherapy should be delayed in case of ANC <1.5x10^9^/L or platelet count of <100 x 10^9^/L. In case of grade I-II neutropenia G-CSF may be used prophylactically to maintain dose intensity.

**Non-hematological toxicities**

| Neurological  NCI grade >2  Sensory alterations or paresthesias interfering with ADL; weakness interfering with ADL; bracing or assistance to walk (e.g. cane or walker).   1. Pretreatment EF, EKG. 2. Evaluate the patient clinically each cycle. If symptoms occur repeat EF. 3. All patients pre-treatment EF and EKG. Repeat EF and EKG every 6 months for 2 years. | Stop treatment |
| --- | --- |
| Cardiac  Decrease in EF <15%  Decrease in EF >15%  Symptomatic chronic heart failure  Asymptomatic bradycardia  Isolated and asymptomatic ventricular  extrasystoles | Reduce treatment by 25%  Stop treatment  Stop treatment  Continue treatment  Continue treatment |
| First degree AV block | Continue treatment under continuous cardiac monitoring |
| Symptomatic arrythmia or  AV block (except 1st degree) | Stop taxane infusion manage arrhythmia according to standard practice |
| Other heart blocks | Patient goes off protocol |
| Other Major Organ Toxicity  (not evaluated as disease related)  Alopecia and nausea NCI grade >2 | Stop treatment  Patient goes off protocol |

Toxic effects are graded using NCI criteria.

##### **Hypersensitivity reactions**

Interruption of taxane infusion

Discontinue taxane infusion for significant hypersensitivity reactions, defined as:

- hypotension requiring pressor therapy

- angioedema

- respiratory distress requiring bronchodilation therapy

- generalized urticaria

For other hypersensitivity reactions, the taxane infusion may be discontinued at the investigator's discretion.

Any significant hypersensitivity reactions and any hypersensitivity reactions requiring treatment discontinuation are to be reported immediately.

The following management of hypersensitivity reactions is recommended:

Administer diphenhydramine 50 mg IV (or its equivalent).

Administer adrenaline (or its equivalent) every 15-20 minutes, until the reaction subsides or a total of six doses are given.

If hypotension is present that does not respond to adrenaline (or its equivalent), administer IV fluids.

If wheezing is present that is not responsive to adrenaline (or its equivalent), administration of nebulized albuterol solution (or its equivalent) is recommended.

Although corticosteroids have no effect on the initial reaction, they have been shown to block "late" allergic reactions to a variety of agents. Thus, methylprednisolone 125 mg IV (or its equivalent) may be administered to prevent recurrent or ongoing allergy manifestations.

**RADIATION THERAPY**

**1. Target volume**

The purpose of breast irradiation is to treat the skin, muscles and lymphatics in the axilla and the supraclavicular fossa (if there are more than 4 positive lymph nodes), the entire surgical scar and any remaining breast tissue. Dose to the underlying lung tissue, cervical spine and brachial plexus should be kept to a minimum.

**2. Treatment technique**

The patient lies on the treatment couch supine, with the ipsilateral arm abducted to 90^0^ and the head away from the affected side. It is advisable to treat all fields with the patient in the same position, in order to avoid overlap at the field junction. Radiotherapy is given with megavoltage machines (either a linear accelerator or a cobalt unit). A treatment planning is prepared for each individual patient, using either a simulator (in this case an outline is taken with plaster or Paris) or CT slices taken in the treatment position.

**3. Field arrangements**

As a standard, a four-field technique excluding internal mammary nodes is applied (two tangential fields, medial and lateral, one anterior and one posterior field covering the axilla and the supraclavicular fossa). The upper border of the tangential fields lies at the level of the manubrium sterni and the lower border 1-2 cm below the margin of the breast tissue. The lateral border lies at least in the midaxillarry line and the medial approximately in the midline. The axilla and supraclavicular fossa are treated using an anterior field whose inferior border lies 0.5 cm above and parallel to the superior border of the tangential fields. The medial border lies 1 cm lateral to the anterior midline. Superiorly, only the supraclavicular nodes are covered. The posterior field covers either the axilla only or both the supraclavicular fossa and the axilla.

As an alternative to the four-field technique, a three-field technique can be applied (two tangential and one anterior-oblique high-axillary supraclavicular field, angled at approximately 10^0^ medially).

Scar extensions, not covered by the standard fields, can be treated by electron beams.

**4. Dose**

**4a.** Chest wall

The dose given to the chest wall is 50-55 Gy. The dose per fraction is in the range of 1.8 to 2.0 Gy. Five fractions are given per week. A dose exceeding 105% (hot spot) is not accepted. Wedges or compensators may be necessary to reduce this hot spot. The underlying tissues should not receive more than 70% of the prescribed dose at a depth of 2 cm.

**4b.** Regional lymph nodes

A total dose of 50-55 Gy is given, calculated at a depth of 1 cm for the supraclavicular nodes. The dose per fraction is in the range of 1.8 to 2.0 Gy. Five fractions are given per week. Radiation of the axilla is not recommended.

**4c.** Βoost technique

An additional dose of 10 Gy is delivered to the tumor bed. The technique depends on the type of operation, i.e. radical mastectomy: 10 MeV electrons; lumpectomy: external beam radiotherapy (photons or electrons) or brachytherapy.
