# Supplementary material for: Dose-dense sequential adjuvant chemotherapy followed, as indicated, by trastuzumab for one year in patients with early breast cancer: first report at 5-year median follow-up of a Hellenic Cooperative Oncology Group randomized phase III trial
Source: BMC Cancer. 2014 Jul 15;14:515. doi: 10.1186/1471-2407-14-515 (PMC4223601; doi:10.1186/1471-2407-14-515)
Supplement: Additional file 2: Table S1 — Treatment compliance and discontinuation reasons. Table S2. Sites of relapse. Table S3. Incidence of adverse events according to treatment arm (as treated). Figure S1. Disease-free survival (A) and overall survival (B) of patients treated in Arm A (E-T-CMF), Arm B (E-CMF-wD) and Arm C (E-CMF-wT). Log-rank p-values are reported. [file 1471-2407-14-515-S2.doc]

**Table S1.** Treatment compliance and discontinuation reasons.

|  | **Arm A**  **E-T-CMF**  ***N*=328** | **Arm B**  **E-CMF-wD**  ***N*=318** | **Arm C**  **E-CMF-wT**  ***N*=344** | **Total study population**  ***N*=990** |
| --- | --- | --- | --- | --- |
|  | **N (%)** | **N (%)** | **N (%)** | **N (%)** |
| Completed treatment (per protocol) | 306 (93.3) | 279 (87.7) | 300 (87.2) | 885 (89.4) |
| Discontinued treatment**^1^** | **22** (6.7) | **39** (12.3) | **44** (12.8) | 105 (10.6) |
| Death as a result of infection | 1 | - | - | 1 |
| Death as a result of pulmonary embolism | - | 1 | 1 | 2 |
| Death as a result of pulmonary respiratory distress | - | 1 | - | 1 |
| Death as a result of myocardial infarction | - | - | 1 | 1 |
| Disease progression | - | 1 | - | 1 |
| Intolerable toxicity | 13 (59.1) | 7 (17.9) | 18 (40.9) | 38 (36.2) |
| Voluntary withdrawal | 4 (18.2) | 20 (51.3) | 14 (31.8) | 38 (36.2) |
| Other reasons | 4 (18.2) | 9 (23.1) | 10 (22.7) | 23 (21.9) |

**^1^***P*=0.004 (Pearson chi-square; Arm A vs. combined B and C Arms).

**Table S2.** Site of relapse.

|  | **Arm A**  **E-T-CMF**  **N=333** | **Arm B**  **E-CMF-wD**  **N=331** | **Arm C**  **E-CMF-wT**  **N=326** | **Total study population**  **N=990** |
| --- | --- | --- | --- | --- |
|  | **N (%)** | **N (%)** | **N (%)** | **N (%)** |
| Locoregional relapse | 2 (0.6) | 10 (3.0) | 5 (1.5) | 17 (0.1) |
| Axillary nodes | - | - | 2 (0.6) | 2 (0.2) |
| Supraclavicular nodes | - | 5 (1.5) | 1 (0.3) | 6 (0.6) |
| Skin | 1 (0.3) | 5 (1.5) | 1 (0.3) | 7 (0.7) |
| Residual breast | 1 (0.3) | 2 (0.6) | 1 (0.3) | 4 (0.4) |
|  |  |  |  |  |
| Distant relapse | 48 (14.4) | 30 (9.1) | 33 (10.1) | 111 (11.2) |
| Brain | 5 (1.5) | 5 (1.5) | 3 (0.9) | 13 (1.3) |
| Bones | 26 (7.8) | 15 (4.5) | 9 (2.8) | 50 (5.0) |
| Lung/Pleura | 17 (5.1) | 9 (2.7) | 13 (4.0) | 39 (3.9) |
| Contralateral breast | 2 (0.6) | - | 1 (0.3) | 3 (0.3) |
| Visceral | 16 (4.8) | 15 (4.5) | 16 (4.9) | 47 (4.7) |
|  |  |  |  |  |

**Table S3.** Incidence of adverse events according to treatment arm (as treated).

|  | Arm A: E-T-CMF | | | | | | | | | | Arm B: E-CMF-wD | | | | | | | | | | **Arm C: E-CMF-wT** | | | | | | | | | |
| --- | --- | --- | --- | --- | --- | --- | --- | --- | --- | --- | --- | --- | --- | --- | --- | --- | --- | --- | --- | --- | --- | --- | --- | --- | --- | --- | --- | --- | --- | --- |
|  | **N=326** | | | | | | | | | | **N=316** | | | | | | | | | | **N=320** | | | | | | | | | |
|  | **Grade I** | | **Grade II** | | **Grade III** | | **Grade IV** | | **Grade V** | | **Grade I** | | **Grade II** | | **Grade III** | | **Grade IV** | | **Grade V** | | **Grade I** | | **Grade II** | | **Grade III** | | **Grade IV** | | **Grade V** | |
|  | N | **%** | N | **%** | N | **%** | N | **%** | N | **%** | N | **%** | N | **%** | N | **%** | N | **%** | N | **%** | N | **%** | N | **%** | N | **%** | N | **%** | N | **%** |
| Hemoglobin | 215 | 66.0 | 54 | 16.6 | 6 | 1.8 |  |  |  |  | 204 | 64.6 | 58 | 18.4 | 3 | 0.9 |  |  |  |  | 202 | 63.1 | 72 | 22.5 | 6 | 1.9 |  |  |  |  |
| Leucocytes | 67 | 20.6 | 84 | 25.8 | 28 | 8.6 | 11 | 3.4 |  |  | 76 | 24.1 | 88 | 27.8 | 36 | 11.4 | 5 | 1.6 |  |  | 104 | 32.5 | 106 | 33.1 | 32 | 10.0 | 7 | 2.2 |  |  |
| Neutrophils | 32 | 9.8 | 57 | 17.5 | 55 | 16.9 | 44 | 13.5 |  |  | 49 | 15.5 | 59 | 18.7 | 61 | 19.3 | 24 | 7.6 |  |  | 65 | 20.3 | 68 | 21.3 | 49 | 15.3 | 36 | 11.3 |  |  |
| Platelets | 35 | 10.7 | 3 | 0.9 | 1 | 0.3 | 1 | 0.3 |  |  | 40 | 12.7 | 3 | 0.9 | 1 | 0.3 | 1 | 0.3 |  |  | 44 | 13.8 | 5 | 1.6 | 3 | 0.9 | 1 | 0.3 |  |  |
| Febrile neutropenia |  |  |  |  | 17 | 5.2 | 2 | 0.6 | 1 | 0.3 |  |  |  |  | 12 | 3.8 | 1 | 0.3 | 1 | 0.3 |  |  |  |  | 13 | 4.1 | 4 | 1.3 |  |  |
| Thrombopenia | 1 | 0.3 |  |  |  |  |  |  |  |  |  |  |  |  |  |  |  |  |  |  |  |  |  |  |  |  |  |  |  |  |
| Gastrointestinal | 58 | 17.8 | 13 | 4.0 |  |  |  |  |  |  | 75 | 23.7 | 32 | 10.1 | 4 | 1.3 |  |  |  |  | 75 | 23.4 | 17 | 5.3 | 4 | 1.3 |  |  |  |  |
| Metabolic/Laboratory | 158 | 48.5 | 44 | 13.5 | 12 | 3.7 |  |  |  |  | 157 | 49.7 | 47 | 14.9 | 7 | 2.2 | 1 | 0.3 |  |  | 136 | 42.5 | 65 | 20.3 | 16 | 5.0 | 5 | 1.56 |  |  |
| Dermatology/Skin | 51 | 15.6 | 30 | 9.2 | 5 | 1.5 |  |  |  |  | 63 | 19.9 | 46 | 14.6 | 9 | 2.8 |  |  |  |  | 60 | 18.8 | 20 | 6.3 | 1 | 0.3 |  |  |  |  |
| Pain | 91 | 27.9 | 77 | 23.6 | 17 | 5.2 |  |  |  |  | 96 | 30.4 | 18 | 5.7 | 3 | 0.9 |  |  |  |  | 100 | 31.3 | 31 | 9.7 | 5 | 1.6 |  |  |  |  |
| Pulmonary/Upper respiratory | 20 | 6.1 | 1 | 0.3 |  |  | 2 | 0.6 |  |  | 29 | 9.2 | 2 | 0.6 | 2 | 0.6 | 1 | 0.3 | 1 | 0.3 | 28 | 8.8 | 1 | 0.3 | 1 | 0.3 |  |  |  |  |
| Constitutional | 53 | 16.3 | 9 | 2.8 |  |  |  |  |  |  | 52 | 16.5 | 15 | 4.7 | 1 | 0.3 |  |  |  |  | 56 | 17.5 | 18 | 5.6 | 1 | 0.3 |  |  |  |  |
| Fatigue | 75 | 23.0 | 42 | 12.9 | 4 | 1.2 |  |  |  |  | 98 | 31.0 | 42 | 13.3 | 7 | 2.2 |  |  |  |  | 86 | 26.8 | 42 | 13.1 | 9 | 2.8 | 1 | 0.3 |  |  |
| Diarrhea | 10 | 3.1 | 9 | 2.8 | 3 | 0.9 |  |  |  |  | 34 | 10.8 | 18 | 5.7 | 8 | 2.5 |  |  |  |  | 32 | 10.0 | 12 | 3.75 | 3 | 0.9 |  |  |  |  |
| Alopecia | 9 | 2.8 | 252 | 77.3 |  |  |  |  |  |  | 11 | 3.5 | 227 | 71.8 |  |  |  |  |  |  | 6 | 1.9 | 259 | 80.9 |  |  |  |  |  |  |
| Nausea | 111 | 34.0 | 78 | 23.9 | 7 | 2.1 |  |  |  |  | 119 | 37.7 | 74 | 23.4 | 6 | 1.9 |  |  |  |  | 122 | 38.2 | 85 | 26.6 | 10 | 3.1 |  |  |  |  |
| Neurology | 113 | 34.7 | 80 | 24.5 | 16 | 4.9 |  |  |  |  | 72 | 22.8 | 15 | 4.7 | 4 | 1.3 |  |  |  |  | 101 | 31.6 | 44 | 13.8 | 4 | 1.3 |  |  |  |  |
| Vomiting | 56 | 17.2 | 43 | 13.2 | 5 | 1.5 |  |  |  |  | 65 | 20.6 | 52 | 16.5 | 5 | 1.6 | 1 | 0.3 |  |  | 73 | 22.8 | 58 | 18.1 | 4 | 1.3 |  |  |  |  |
| Mucositis | 32 | 9.8 | 15 | 4.6 | 6 | 1.8 | 1 | 0.3 |  |  | 49 | 15.5 | 32 | 10.1 | 17 | 5.4 |  |  |  |  | 47 | 14.7 | 21 | 6.6 | 10 | 3.1 |  |  |  |  |
| Infection | 11 | 3.4 | 18 | 5.5 | 7 | 2.1 |  |  | 1 | 0.3 | 10 | 3.2 | 21 | 6.6 | 15 | 4.7 |  |  |  |  | 13 | 4.1 | 44 | 13.8 | 7 | 2.2 |  |  |  |  |
| Occular | 11 | 3.4 | 3 | 0.9 |  |  |  |  |  |  | 59 | 18.7 | 15 | 4.7 | 2 | 0.6 |  |  |  |  | 19 | 5.9 | 9 | 2.8 |  |  |  |  |  |  |
| Allergy | 24 | 7.4 | 19 | 5.8 | 8 | 2.5 |  |  |  |  | 22 | 7.0 | 4 | 1.3 |  |  |  |  |  |  | 29 | 9.1 | 6 | 1.9 |  |  | 2 | 0.6 |  |  |
| Hemorrhage | 3 | 0.9 | 2 | 0.6 |  |  |  |  |  |  | 33 | 10.4 | 7 | 2.2 |  |  |  |  |  |  | 20 | 6.3 | 2 | 0.6 |  |  |  |  |  |  |
| Edema | 6 | 1.8 |  |  |  |  |  |  |  |  | 15 | 4.7 | 5 | 1.6 | 1 | 0.3 |  |  |  |  | 24 | 7.5 | 2 | 0.6 |  |  |  |  |  |  |
| Vascular | 1 | 0.3 | 4 | 1.2 | 2 | 0.6 |  |  |  |  | 3 | 0.9 | 6 | 1.9 | 3 | 0.9 |  |  |  |  | 1 | 0.3 | 15 | 4.7 | 7 | 2.2 |  |  | 1 | 0.3 |
| Cardiac | 8 | 2.5 | 2 | 0.6 |  |  | 1 | 0.3 |  |  | 12 | 3.8 | 3 | 0.9 | 2 | 0.6 |  |  |  |  | 9 | 2.8 | 1 | 0.3 | 1 | 0.3 |  |  | 1 | 0.3 |
| Lymphopenia | 5 | 1.5 |  |  | 2 | 0.6 |  |  |  |  | 3 | 0.9 | 1 | 0.3 | 4 | 1.3 |  |  |  |  | 6 | 1.9 | 7 | 2.2 | 1 | 0.3 |  |  |  |  |
| Anorexia | 1 | 0.3 |  |  |  |  |  |  |  |  | 7 | 2.2 | 2 | 0.6 | 1 | 0.3 |  |  |  |  | 1 | 0.3 | 2 | 0.6 |  |  |  |  |  |  |
| Sexual |  |  | 1 | 0.3 |  |  |  |  |  |  | 3 | 0.9 | 2 | 0.6 |  |  |  |  |  |  | 2 | 0.6 | 1 | 0.3 |  |  |  |  |  |  |
| Herpes zoster | 1 | 0.3 |  |  |  |  |  |  |  |  | 1 | 0.3 | 2 | 0.6 |  |  |  |  |  |  | 1 | 0.3 | 2 | 0.6 |  |  |  |  |  |  |
| Renal | 1 | 0.3 |  |  |  |  |  |  |  |  | 1 | 0.3 | 2 | 0.6 |  |  |  |  |  |  | 1 | 0.3 | 1 | 0.3 |  |  |  |  |  |  |
| Flu like syndrome | 5 | 1.5 |  |  |  |  |  |  |  |  |  |  |  |  |  |  |  |  |  |  |  |  |  |  |  |  |  |  |  |  |
| Musculoskeletal | 1 | 0.3 |  |  |  |  |  |  |  |  | 2 | 0.6 | 1 | 0.3 |  |  |  |  |  |  |  |  |  |  |  |  |  |  |  |  |
| Auditory |  |  |  |  |  |  |  |  |  |  | 1 | 0.3 |  |  |  |  |  |  |  |  |  |  | 2 | 0.6 |  |  |  |  |  |  |
| Lymphatics | 1 | 0.3 |  |  |  |  |  |  |  |  |  |  |  |  |  |  |  |  |  |  | 2 | 0.6 |  |  |  |  |  |  |  |  |
| Endocrine |  |  |  |  |  |  |  |  |  |  | 2 | 0.6 |  |  |  |  |  |  |  |  |  |  |  |  |  |  |  |  |  |  |

**Figure S1.** Disease-free survival (A) and overall survival (B) of patients treated in Arm A (E-T-CMF), Arm B (E-CMF-wD) and Arm C (E-CMF-wT). Log-rank p-values are reported.

| **A.** | **B.** |
| --- | --- |
|  |  |
